# Effect of Lecithin-Bound Iodine Treatment on Inherited Retinal Degeneration in Mice

**DOI:** 10.1167/tvst.10.13.8

**Published:** 2021-11-09

**Authors:** Hideo Kohno, Ryo Terauchi, Sumiko Watanabe, Kosuke Ichihara, Tomoyuki Watanabe, Euido Nishijima, Akira Watanabe, Tadashi Nakano

**Affiliations:** 1 Department of Ophthalmology, The Jikei University School of Medicine, Tokyo, Japan; 2 Department of Retinal Biology and Pathology, Graduate School of Medicine, The University of Tokyo, Tokyo, Japan

**Keywords:** lecithin-bound iodine, retinal degeneration, microglia, macrophage, CCL2, CCR2

## Abstract

**Purpose:**

Although lecithin-bound iodine (LBI) has been administered orally for retinal diseases, a lack of clinical studies and obscure action mechanism of LBI hinder its large-scale prescription. LBI treatment suppresses chemokine (C-C motif) ligand 2 (CCL2) secretion from retinal pigment epithelial cells *in vitro*. Herein, we assessed the in vivo effect of LBI treatment on retinal degeneration (RD) in mice.

**Methods:**

*Mertk^−/−^Cx3cr1^GFP/+^Ccr2^RFP/+^* mice—a model for RD—demonstrate fluorescein-labeled microglia/macrophage to facilitate visualization of CX3CR1-green fluorescent protein (GFP) and CCR2-red fluorescent protein (RFP). An LBI-containing mouse diet was provided to *Mertk^−/−^Cx3cr1^GFP/+^Ccr2^RFP/+^* mice ad libitum from postnatal day (POD) 28. CX3CR1-GFP and CCR2-RFP expression was assessed at POD 56 using retinal sectioning and flat mounting. RD severity was assessed at POD 84. Retinal RNA was extracted from the mice of each group to measure chemokine expression. Electroretinography was performed to assess retinal function.

**Results:**

CCR2-RFP expression in the retina and retinal pigment epithelial cells was suppressed by LBI treatment compared with that in the control at POD 56. The number of outer nuclear layer nuclei was higher in the group fed with LBI-containing diet than in the control mice at POD 84. *Ccl2* and *Ccr2* RNA expression was suppressed by LBI intake. Electroretinography showed the LBI-treated group to have a high b-wave amplitude compared with the control group.

**Conclusions:**

Suppressing CCR2-RFP–positive macrophage invasion into the retina and CCL2 and CCR2 expression is a potential mechanism underlying LBI-mediated attenuation of RD.

**Translational Relevance:**

Life-long LBI administration may become a candidate for treating RD.

## Introduction

Retinal degeneration (RD), including retinitis pigmentosa and age-related macular degeneration, is a leading cause of blindness.^1^^,^^2^ Although photoreceptor cell death is responsible for RD, several studies have provided evidence regarding involvement of inflammation in RD progression.^3^^–^^5^ Intravitreal anti-vascular endothelial growth factor therapeutics are widely used in treating patients with wet age-related macular degeneration. However, anti-vascular endothelial growth factor therapeutics cannot rescue photoreceptor cell death. Therefore, inflammation is now being considered a potential therapeutic target in RD.^6^^,^^7^ With the onset of degeneration, both the mechanisms underlying primary cell death (e.g., genetic abnormalities and aging) and the inflammatory cell death cascade are activated. To eliminate dead photoreceptor cell debris, phagocytic cells, including microglia, and retinal pigment epithelium (RPE) cells are required.^3^^,^^8^^,^^9^ If resident phagocytic cells cannot afford to eliminate the cellular debris, they secrete cytokines and chemokines to induce the infiltration of monocyte-derived macrophages into the retina via the broken blood–retinal barrier.^10^ The macrophage infiltration drives inflammation, which in turn, accelerates RD and can even lead to blindness.^11^^,^^12^

To maintain retinal homeostasis and preserve photoreceptor cells, harmful retinal inflammation must be controlled. Studies are now focusing on methods to control retinal inflammation, and this approach holds promise for RD treatment.^13^^–^^15^ However, this approach should have minimum side effects because RD requires life-long therapy. In Japan, lecithin-bound iodine (LBI), considered an “old” drug, has been administered clinically for retinal diseases, including central serous chorioretinopathy, which causes serous retinal detachment (only Japanese reports). LBI was originally developed in 1955 for treating Graves’ disease in Japan. The administration of iodine agents or thyroid hormone agents was believed to enhance retinal metabolism.^16^^,^^17^ The efficacy of LBI against retinal diseases was assessed in Japan (only Japanese reports); in the 1950s, LBI was widely prescribed for retinal diseases, including central serous chorioretinopathy, age-related macular degeneration, retinal detachment, and vitreous hemorrhage. However, with recent medical advancements and the development of surgical techniques, the frequency of LBI prescription has been decreasing steadily. Currently, LBI is prescribed mainly for patients with central serous chorioretinopathy. Recently, Sugimoto et al.^18^ reported that LBI administration prevents hypoxic damage to RPE cells and suppresses CCL2 secretion from RPE cells in vitro. Elevation of the chemokine (C-C motif) ligand 2 (CCL2) expression is a common feature of inflammatory response in RD.^19^ The expression of CCL2 in the retina and RPE is negligible in healthy young adult animals, but increases in acute inflammation and degeneration.^20^ We hypothesized that the LBI-mediated prevention of inflammatory response in retina may attenuate RD in vivo. To test this hypothesis, in this study, we focused on LBI as a treatment agent for RD. For this, we used *Mertk^−/−^Cx3cr1^GFP/+^Ccr2^RFP/+^* mice.^21^ This model develops RD owing to a lack of phagocytosis of photoreceptor outer segments by the RPE cells; in addition, CX3CR1 and CCR2 are labeled with fluorescein, which enables the observation of CX3CR1-positive microglia migration and CCR2-positive macrophage infiltration.^22^

In this study, we demonstrated that LBI treatment ameliorated RD and suppressed CCR2-RFP expression in *Mertk^−/−^Cx3cr1^GFP/+^Ccr2^RFP/+^* mice. Additionally, RNA levels of *Ccl2* and *Ccr2* were suppressed in the LBI-treated group. Our study provides new insights on use of LBI as a safe drug option for life-long RD therapy.

## Methods

### Animals

*Mertk^−/−^Cx3cr1^GFP/+^Ccr2^RFP/+^* mice and *Mertk-^+/+^Cx3cr1^GFP/+^Ccr2^RFP/+^* mice were generated as previously described.^21^ *Mertk^+/+^Cx3cr1^GFP/+^Ccr2-^RFP/+^* mice (littermate of *Mertk^−/−^Cx3cr1^GFP/+^Ccr2-^RFP/+^* mice) were used as the negative controls. Genotyping was performed as described previously.^21^

Equal numbers of male and female mice were used in this study. All mice were housed in the animal facility at the Jikei University School of Medicine, where they were maintained under pathogen-free conditions either in complete darkness or a 12-hour light (∼10 lux)/12-hour dark cycle. Standard animal cages (42.5 × 26.6 × 19 cm) placed on solid a floor were used. Mice of the same gender were housed socially. All animal procedures and experiments were approved by the Jikei University School of Medicine Animal Care Committees (approval number: #2020-53) and conformed with the recommendations of both the American Veterinary Medical Association Panel on Euthanasia and the ARVO Statement for the Use of Animals in Ophthalmic and Vision Research.

### LBI Administration to the Mice

Mice were provided with two types of LBI (Jolethin, Daiichi Pharmaceutical Co., Tokyo, Japan)-supplemented animal feed: high (0.00112%; LBI-high) and low (0.00037%; LBI-low). Control feed was prepared based on AIN-93G mouse food formulations published by the American Institute of Nutrition committee.^23^ The LBI-supplemented feed (LBI-high or LBI-low) or control diet was provided to the mice from just after weaning (postnatal day [POD] 28) until evaluation.

### Animal Numbers

**Figure 1.**
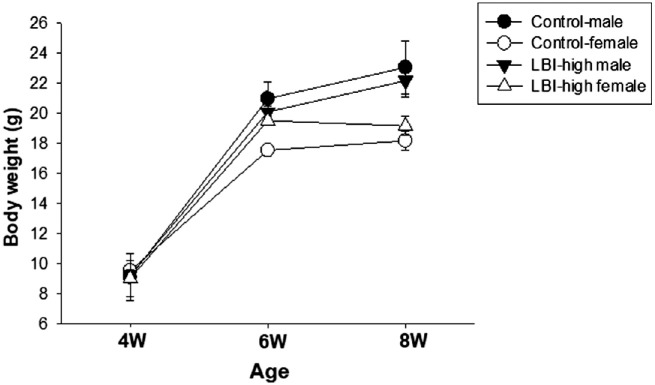
Preparation of LBI-supplemented mouse feed. Two types of LBI-supplemented mouse feed (0.00112% LBI, LBI-high; and 0.00037% LBI, LBI-low) were prepared. Feed without LBI was prepared as a control. Detailed nutrients are described in the Table. Body weight gain of LBI-high or control mice is shown.

**Figure 2.**
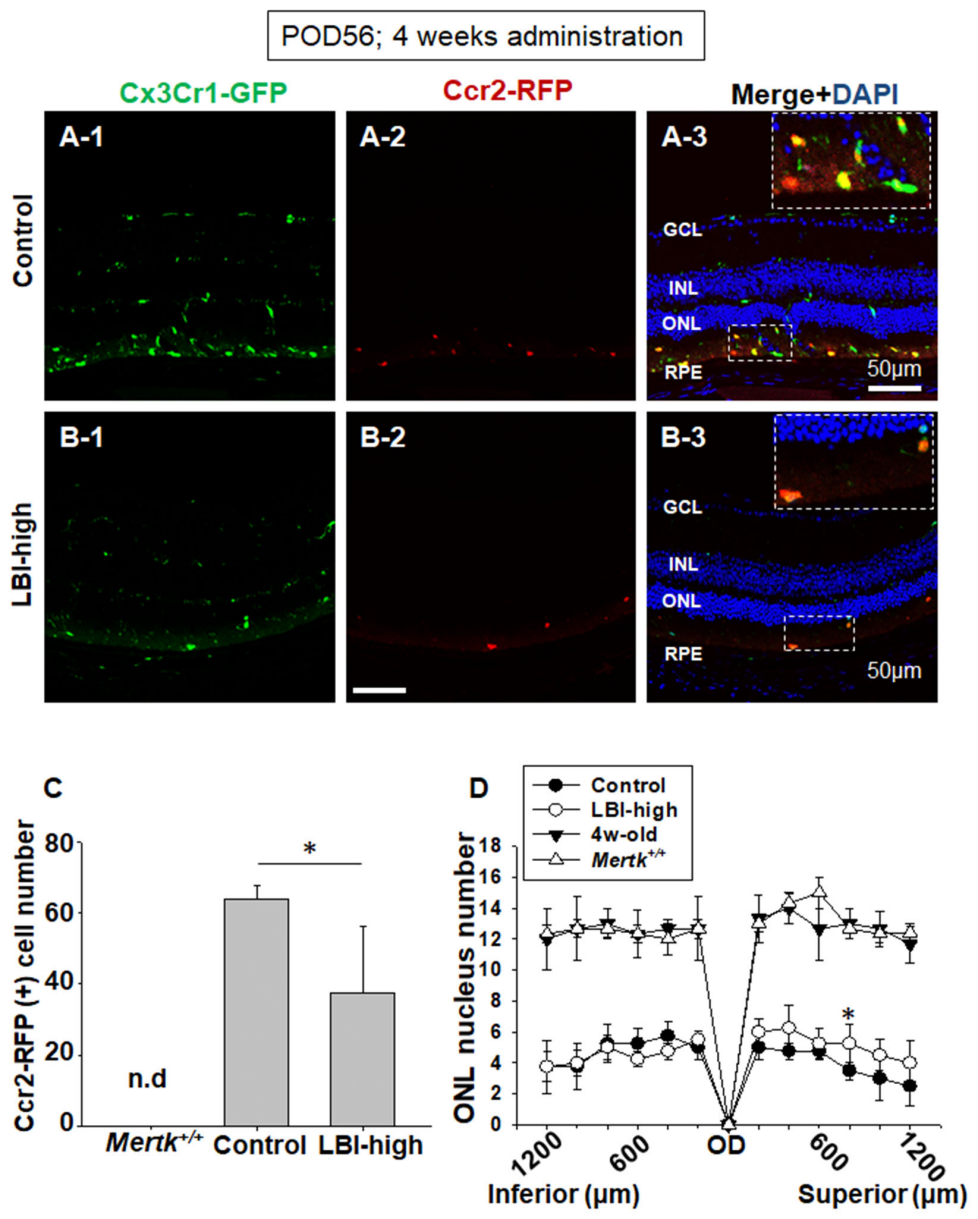
LBI-administered *Mertk^−/−^Cx3cr1^GFP/+^Ccr2^RFP/+^* mice showed suppression of CCR2-RFP expression. Retinal sections of LBI-high (A) and control (B) *Mertk^−/−^Cx3cr1^GFP/+^Ccr2^RFP/+^* mice are shown. CCR2-RFP expression was suppressed in the LBI-high group compared with that in the control group (A-2 and B-2). CX3CR1-GFP and CCR2 merged with DAPI staining are shown (A-3 and B-3). Representative magnified CX3CR1-GFP– and CCR2-RFP–positive cells in the subretinal space are shown in the inset (dashed rectangle). CCR2-RFP–positive cells from the OD to inferior ciliary body (CB) were counted; LBI-high *Mertk^−/−^Cx3cr1^GFP/+^Ccr2^RFP/+^* mice showed significant suppression compared with the control group (C). No CCR2-RFP–positive cells were observed in *Mertk^+/+^Cx3cr1^GFP/+^Ccr2^RFP/+^* mice (*Mertk^+/+^*). n.d., not detected. The number of ONL nuclei was counted every 200 µm from the OD to inferior or superior retina. LBI-high *Mertk^−/−^Cx3cr1^GFP/+^Ccr2^RFP/+^* mice showed marginally retained but similar ONL nuclei number compared with the controls at POD 56. The ONL nuclei number of *Mertk^+/+^Cx3cr1^GFP/+^Ccr2^RFP/+^* (*Mertk^+/+^*) and 4-week-old *Mertk^−/−^Cx3cr1^GFP/+^Ccr2^RFP/+^* mice are shown as negative control. *P < 0.05.

**Figure 3.**
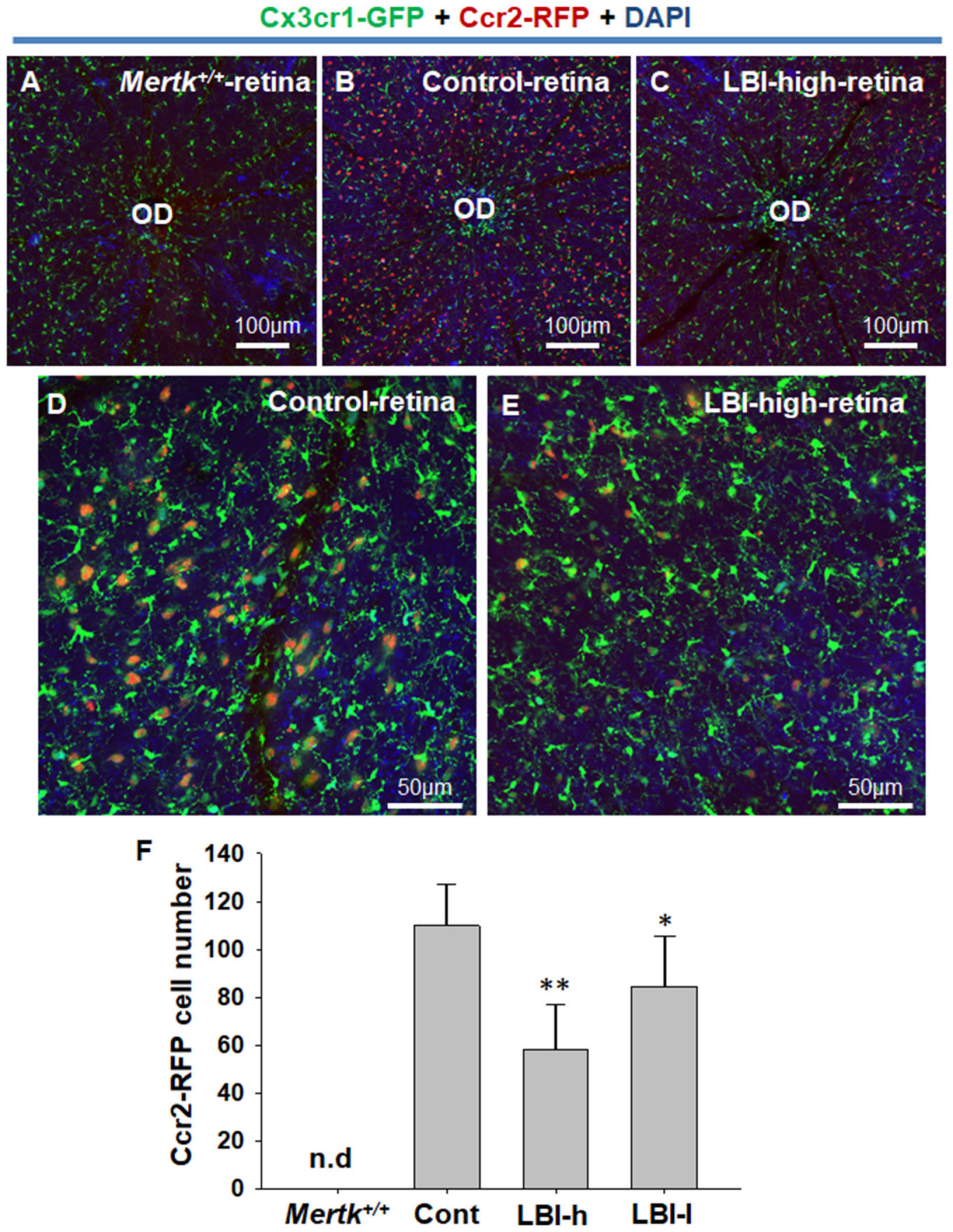
CCR2-RFP expression was suppressed by LBI administration. Retinal flat-mounting of LBI-high (B and D) or control (C and E) *Mertk^−/−^Cx3cr1^GFP/+^Ccr2^RFP/+^* mice was performed. Retinal flat mount of *Mertk^+/+^Cx3cr1^GFP/+^Ccr2^RFP/+^* mice (*Mertk^+/+^*) is shown as negative control (A). CCR2-RFP–positive cells of each group (*Mertk^+/+^*, *n* = 4; Control [Cont], *n* = 12; LBI-high [LBI-h], *n* = 8; and LBI-low [LBI-l], *n* = 8) in field (∼1.8 × 10^−7^ m^2^) were counted. *P < 0.05 compared with control group. Separate CCR2-RFP and CX3CR1-GFP images are shown in Supplementary Figure S2.

**Figure 4.**
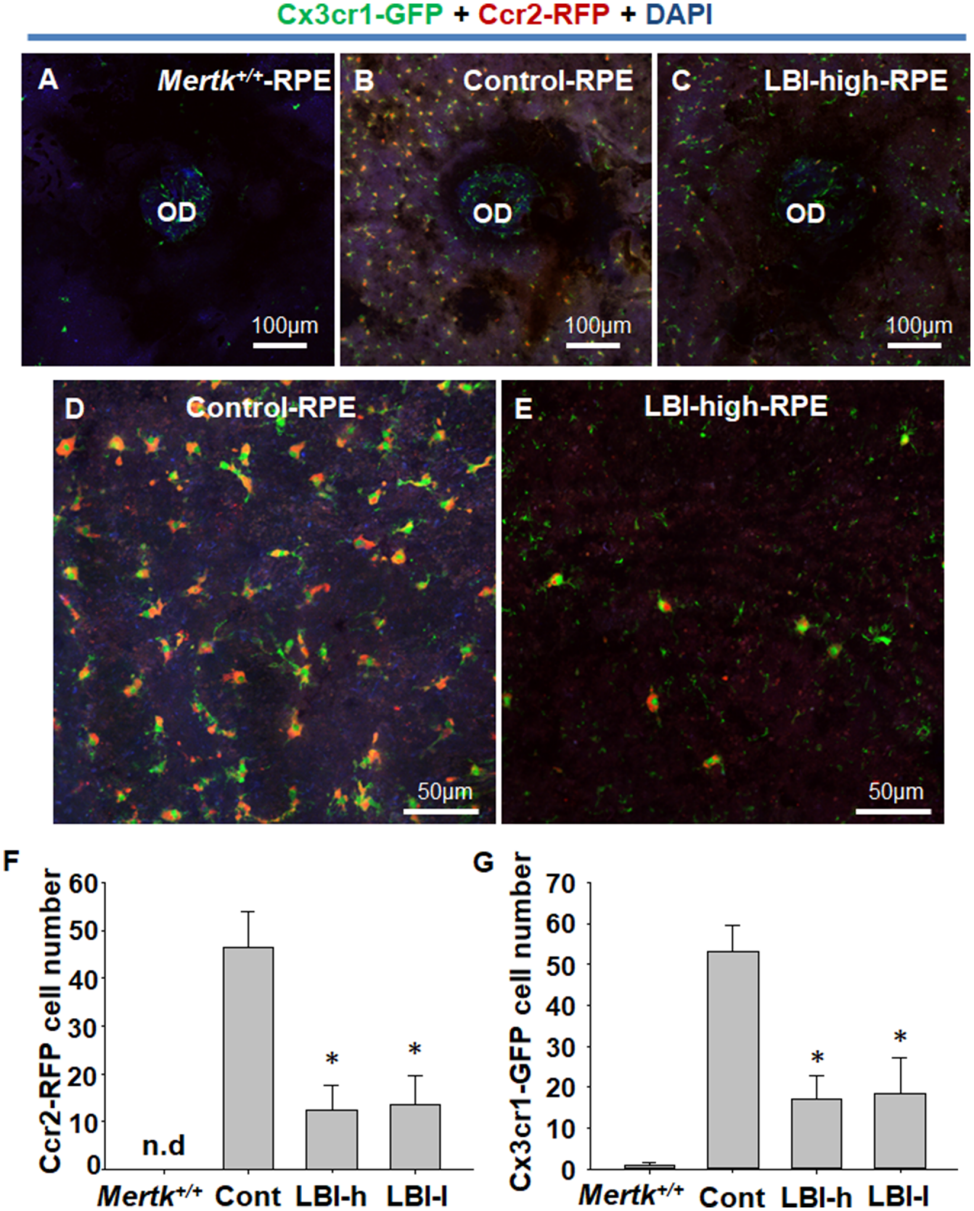
CCR2-RFP– and CX3CR1-GFP–positive cell migration to subretinal space was suppressed by LBI administration. RPE flat mounting was performed to precisely observe the RPE and subretinal space. RPE flat mount of *Mertk^+/+^Cx3cr1^GFP/+^Ccr2^RFP/+^* mice (*Mertk^+/+^*) is shown as negative control (A). CX3CR1-GFP expression was observed mainly at the OD, and only faint expression was observed at the RPE (A). Control *Mertk^−/−^Cx3cr1^GFP/+^Ccr2^RFP/+^* mice showed CCR2-RFP– and CX3CR1-GFP–positive cell migration to the RPE and subretinal space (B and D). LBI-high *Mertk^−/−^Cx3cr1^GFP/+^Ccr2^RFP/+^* mice showed suppression of CCR2-RFP- and CX3CR1-GFP-positive cell migration to the RPE and subretinal space (C and E). CCR2-RFP– and CX3CR1-GFP–positive cells of each group (*Mertk^+/+^*, *n* = 4; Control [Cont], *n* = 12; LBI-high [LBI-h], *n* = 8; and LBI-low [LBI-l], *n* = 8) were counted. *P < 0.05 compared with the control group. Separate CCR2-RFP and CX3CR1-GFP images are shown in Supplementary Figure S3.

**Figure 5.**
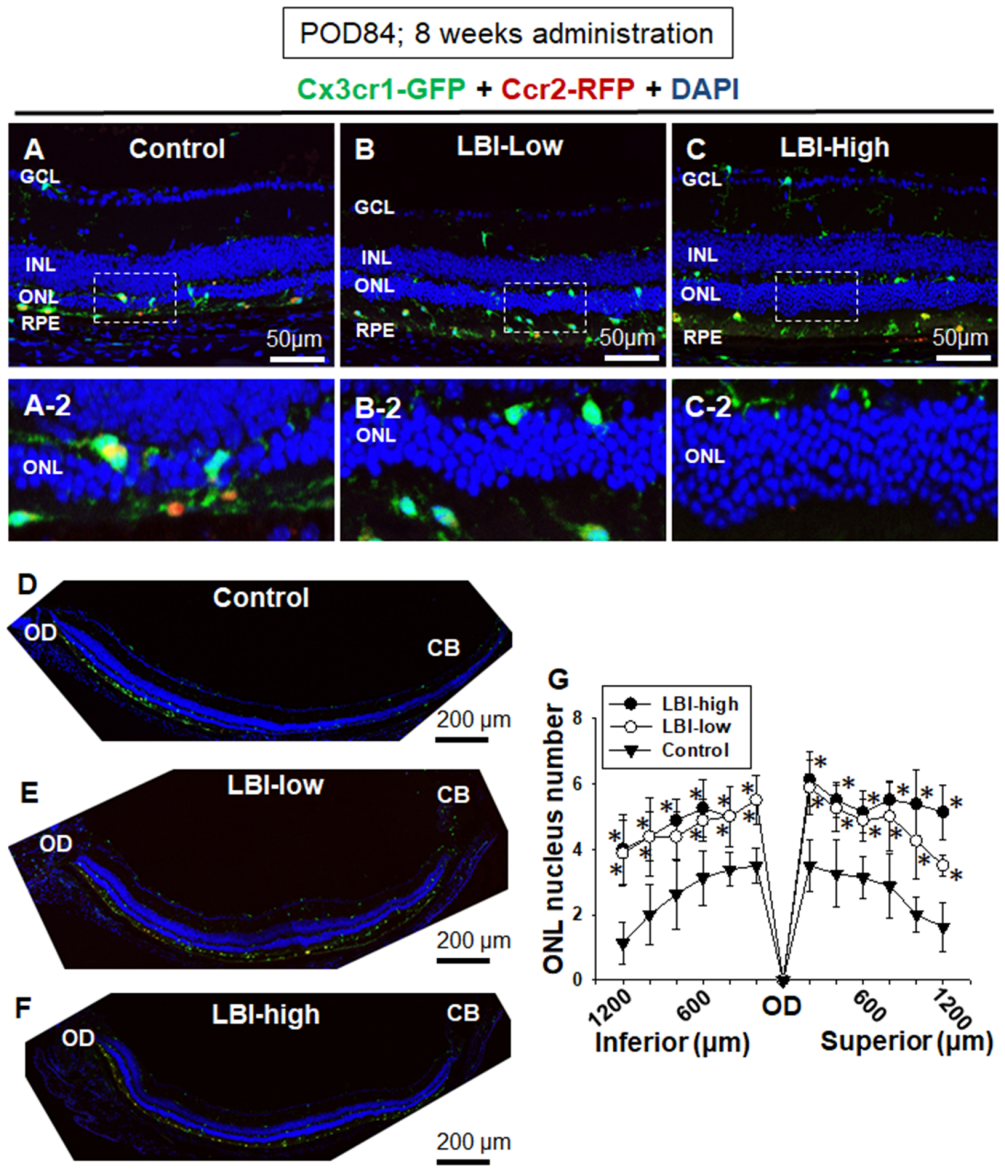
LBI-administered *Mertk^−/−^Cx3cr1^GFP/+^Ccr2^RFP/+^* mice showed attenuation of RD at POD 84. LBI-high, LBI-low, and control feed were provided to the mice until POD 84. Severity of RD was assessed using retinal sectioning. Control *Mertk^−/−^Cx3cr1^GFP/+^Ccr2^RFP/+^* mice showed severe RD at POD 84 (A and D). Magnified image of the ONL and subretinal space is shown (A-2). Retinal sections of LBI-high (C) and LBI-low (B) *Mertk^−/−^Cx3cr1^GFP/+^Ccr2^RFP/+^* mice are shown. Preserved nuclei in ONL were observed in LBI-high (C-2) and LBI-low (B-2) groups compared with those in the control group (A-2). Entire inferior retina (from the OD to ciliary body [CB]) are shown (D, E, and F). ONL nuclei number of each 200 from OD to inferior or superior retina are shown (G). LBI-high (*n* = 10) and LBI-low (*n* = 10) groups showed significant attenuation of RD compared with the control group (*n* = 12). *P < 0.05 compared with the control group.

**Figure 6.**
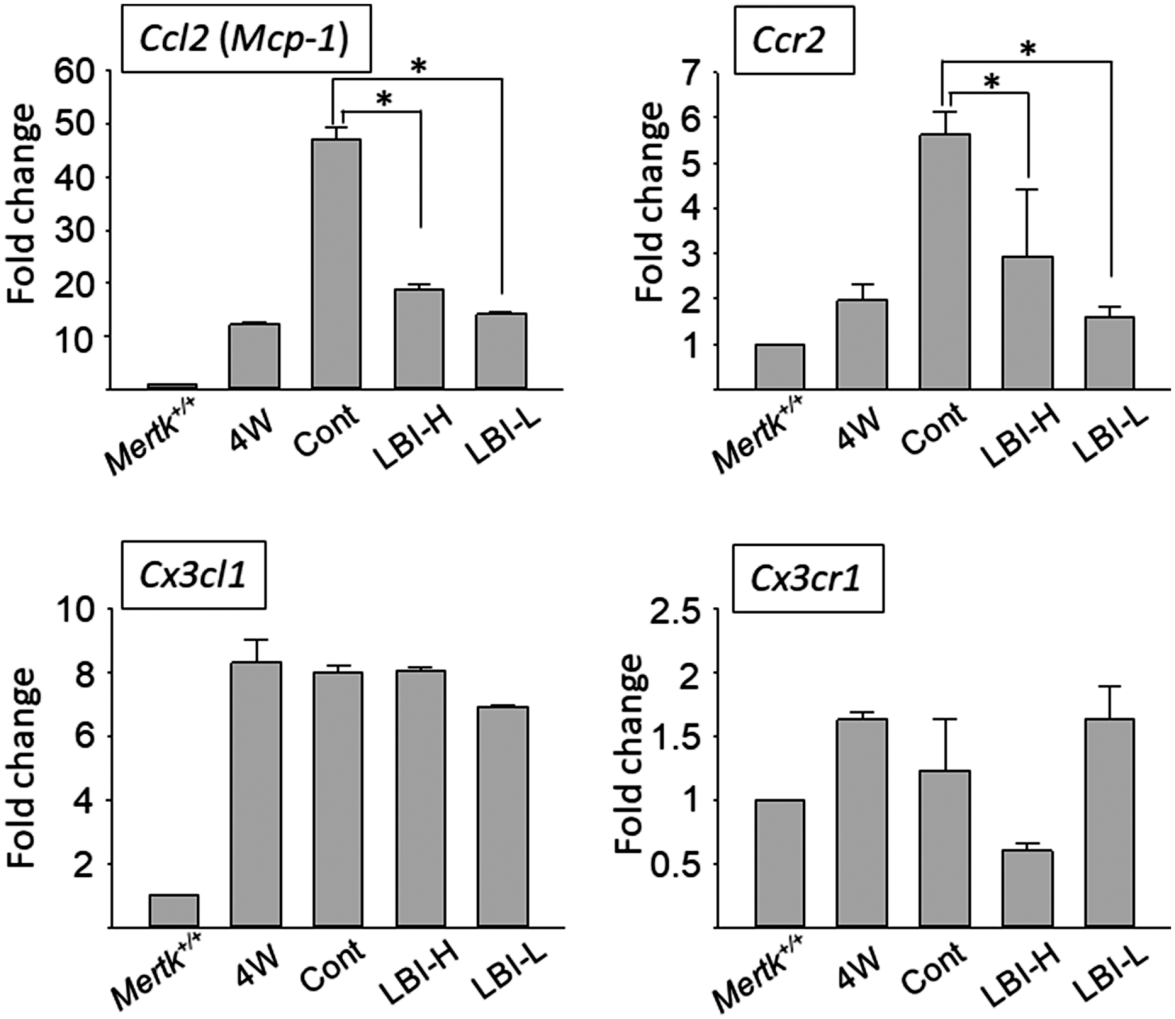
*Ccl2* and *Ccr2* RNA expression levels decreased in LBI-administered *Mertk^−/−^Cx3cr1^GFP/+^Ccr2^RFP/+^* mice. RNA was harvested from retinas of *Mertk^+/+^Cx3cr1^GFP/+^Ccr2^RFP/+^* mice (*Mertk^+/+^*) (*n* = 12) and 4-week-old *Mertk^−/−^Cx3cr1^GFP/+^Ccr2^RFP/+^* mice (*n* = 12) as negative controls. Control (cont) (*n* = 12), LBI-high (LBI-H) (*n* = 16), and LBI-low (LBI-l) (*n* = 12) were provided to mice POD 28–42 for 2 weeks. RNA was harvested from retinas of each group at POD 42. Each RNA expression level was normalized against expression of the housekeeping gene *Gapdh*. The level of *Mertk^+/+^* was adjusted to 1. Fold changes in each group are shown. Error bars indicate mean ± standard deviation (S.D). *P < 0.05.

**Figure 7.**
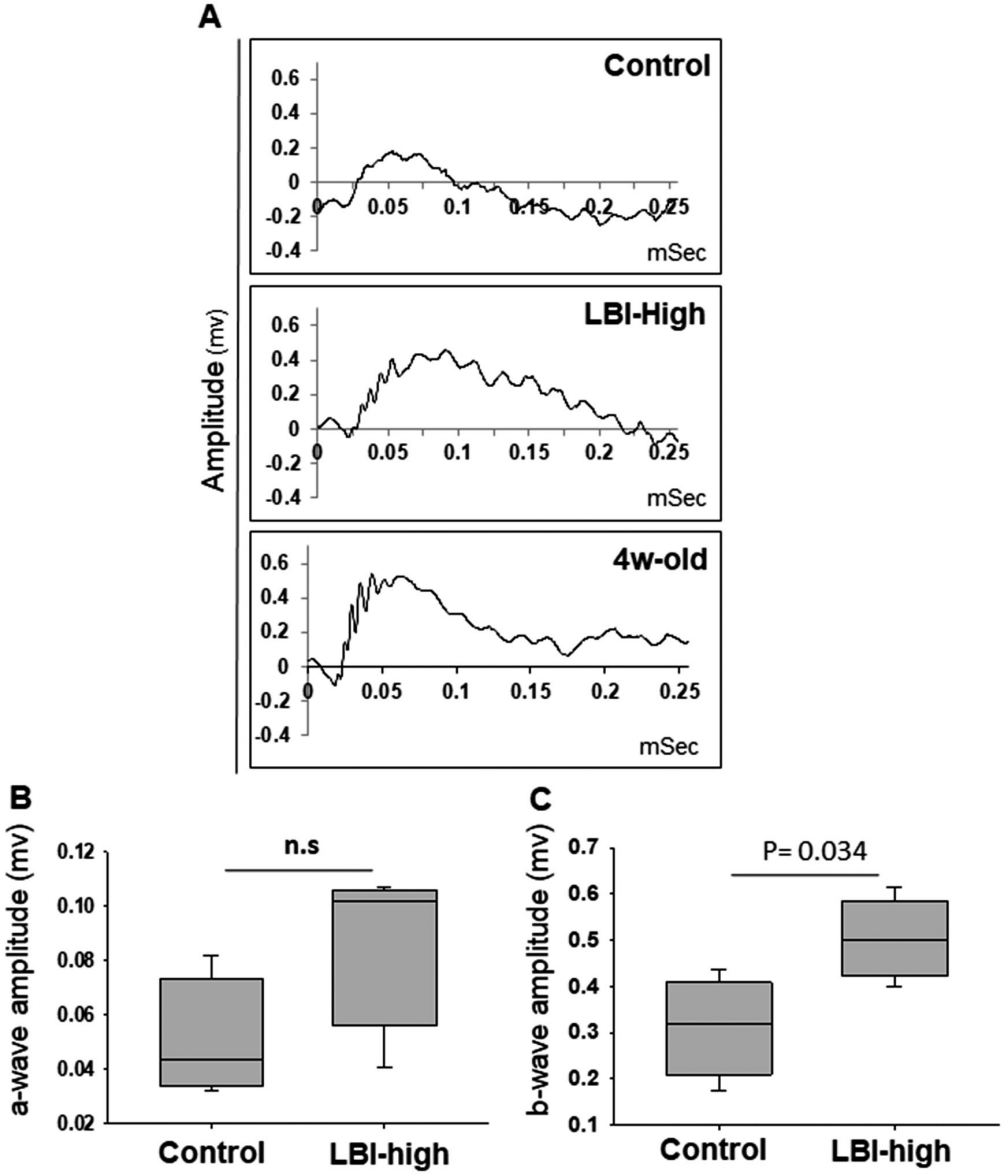
ERG showed partial retinal function preservation in the LBI treatment group compared with the control group. Representative ERG responses at scotopic conditions for the control, LBI-high, and 4-week-old *Mertk^−/−^Cx3cr1^GFP/+^Ccr2^RFP/+^* mice are shown (A). ERG a-wave (B) and b-wave (C) amplitudes of the control (*n* = 4) and LBI-high (*n* = 4) groups are shown in boxplots. n.s., not significant.

To assess the mice body weight for recording adverse events (Fig. 1), LBI-high and control groups included five male and five female *Mertk^−/−^Cx3cr1^GFP/+^Ccr2^RFP/+^* mice. For the 4-week LBI administration study (Fig. 2), the control (*n* = 5) and LBI-high (*n* = 5) groups included *Mertk^−/−^Cx3cr1^GFP/+^Ccr2^RFP/+^* mice. *Mertk^+/+^Cx3cr1^GFP/+^Ccr2^RFP/+^* mice (*n* = 3) and 4-week-old *Mertk^−/−^Cx3cr1^GFP/+^Ccr2^RFP/+^* mice (*n* = 3) were used as negative controls. For the flat mount study (Figs. 3 and 4), the control (*n* = 12), LBI-high (*n* = 8), and LBI-low (*n* = 8) groups included *Mertk^−/−^Cx3cr1^GFP/+^Ccr2^RFP/+^* mice. For the 8-week LBI administration study (Fig. 5), the control (*n* = 12), LBI-high (*n* = 10), and LBI-low (*n* = 10) groups included *Mertk^−/−^Cx3cr1^GFP/+^Ccr2^RFP/+^* mice. For the RNA expression study (Fig. 6), the control (*n* = 5), LBI-high (*n* = 5), and LBI-low (*n* = 5) groups included *Mertk^−/−^Cx3cr1^GFP/+^Ccr2^RFP/+^* mice. *Mertk^+/+^Cx3cr1^GFP/+^Ccr2^RFP/+^* mice (*n* = 5) and 4-week-old *Mertk^−/−^Cx3cr1^GFP/+^Ccr2^RFP/+^* mice (*n* = 5) were used as negative controls. For the electroretinography (ERG) study (Fig. 7), the control (female, *n* = 4) and LBI-high (female, *n* = 4) groups included *Mertk^−/−^Cx3cr1^GFP/+^Ccr2^RFP/+^* mice. Four-week-old *Mertk^−/−^Cx3cr1^GFP/+^Ccr2^RFP/+^* mice (female, *n* = 2) were used as negative controls.

### Retina and RPE Flat Mounting

Flat mount is a suitable technique to count CCR2-RFP– and CX3CR1-GFP–positive cells in *Mertk^−/−^Cx3cr1^GFP/+^Ccr2^RFP/+^* mice.^7^^,^^21^ All procedures for the retina and RPE flat mounting were carried out as described previously.^3^ Briefly, enucleated eyeballs were fixed in 4% paraformaldehyde for 10 minutes. The cornea, lens, and optic nerve were harvested under a surgical microscope to make an eye cup; vertical cutting was flat mounted. The retina was then carefully peeled from the RPE to create a retina flat mount and RPE flat mount under a surgical microscope. Images of flat mounts were captured using a confocal microscope (LSM880, Carl Zeiss, Thornwood, NY). For retina flat mounting, the entire retina was captured at 5-µm intervals, and all photographs were projected in one slice. For RPE flat mounting, the entire visible RPE was captured at 3-µm intervals and projected in one slice. The wavelengths of the laser used for visualization of fluorescent signals are as follows: Cx3cr1-GFP; excitation wavelength 488 nm, emission wavelength 532 nm, Ccr2-RFP; excitation wavelength 633 nm, emission wavelength 696 nm, DAPI; excitation wavelength 405 nm, emission wavelength 450 nm.

### Histologic Analysis

Retinal sections were prepared as previously described.^3^^,^^24^ CCR2-red fluorescent protein (RFP)- and CX3CR1-green fluorescent protein (GFP)–positive cell number was counted either using ImageJ (National Institutes of Health, Bethesda, MD) or manually. Immunohistology images were captured using a confocal microscope (LSM880). The outer nuclear layer (ONL) nuclei number of retinal sections including superior and inferior ciliary body and the optic disc (OD) were counted using ImageJ. Distance from the OD was measured using the image software ZEISS ZEN (Carl Zeiss). The number of ONL nuclei at every 200 µm from the OD was counted manually.

### Quantitative Reverse-Transcription Polymerase Chain Reaction

Retina samples from each group were collected from 10 eyeballs. Total RNA was isolated using RiboPure Kit (Applied Biosystems, Austin, TX), and cDNA was synthesized using SuperScript II Reserve Transcriptase (Invitrogen, Waltham, MA) following the manufacturer's instructions. Real-time polymerase chain reaction amplification was performed using TB Green Premix Ex Taq II (Takara Bio Inc. Shiga, Japan) according to manufacturer's instructions. Quantitative reverse-transcription polymerase chain reaction primers for *Ccl2*, *Ccr2*, *Cx3cl1*, *Cx3cr1,* and the housekeeping gene *Gapdh* were purchased from Takara Bio Inc. Relative expression of genes was normalized to the expression of *Gapdh*.

### ERG

ERG was performed using LS-W (Mayo Corporation, Inazawa, Japan) with LabChart 7 software (ADInstruments, Dunedin, New Zealand). Mice were anesthetized by intramuscular injection of a ketamine–xylazine mixture (50 and 10 mg/kg, respectively). One drop of phenylephrine (1%) was applied for pupil dilation. Scotopic electroretinograms were recorded with increasing intensities of light flashes in the dark-adapted state (>12 h). Flashes were presented at 30- to 60-second intervals. Three to four trials were averaged for single-flash responses. Thirty-two trials were averaged for single-flash responses.

### Data Analysis

Data are presented as the mean ± standard deviation. Results were obtained from at least three independent experiments. The groups were compared using one-way analysis of variance with the SigmaPlot software (Systat Software, Inc, San Jose, CA). A *P* value of less than 0.05 was considered statistically significant.

## Results

### Preparation and Characterization of LBI-Supplemented Feed

The LBI-supplemented animal feed was prepared. To test the possibility of adverse events, the mice that were fed LBI-high and control diets were weighed and compared. The body weights of LBI-high– and control feed–administered male and female *Mertk^−/−^Cx3cr1^GFP/+^Ccr2^RFP/+^* mice are shown in Figure 1. Both groups showed similar weight gain, indicating that they could tolerate and digest the LBI-supplemented feed. A detailed nutrient content of each feed is shown in the Table.

**Table.** Detailed Nutrient Content of Control, LBI-High and LBI-Low Feed. Detailed Nutrient Content of LBI-Supplemented or Nonsupplemented Feed Are Shown

		Control		LBI-Low		LBI-High	
	Product	g%	kcal%	g%	kcal%	g%	kcal%
	Protein	20	20	20	20	20	20
	Carbohydrate	64	64	64	64	64	64
	Fat	7	16	7	16	7	16
	Total		100		100		100
	Kcal/g	4.0		4.0		4.0	
kcal/g	Ingredient	g	kcal	g	kcal	g	kcal
4	Casein	200	800	200	800	200	800
4	L-Cystine	3	12	3	12	3	12
4	Corn starch	397.486	1590	397.486	1590	397.486	1590
4	Maltodextrin 10	132	528	132	528	132	528
4	Sucrose	100	400	100	400	100	400
0	Cellulose, BW200	50	0	50	0	50	0
9	Soybean oil	70	630	70	630	70	630
0	t-Butylhydroquinone	0.014	0	0.014	0	0.014	0
0	Mineral mix S10022G	35	0	35	0	35	0
4	Vitamin mix V10037	10	40	10	40	10	40
0	Choline bitartrate	2.5	0	2.5	0	2.5	0
	LBI	0	0	0.0037	0	0.0112	0
	Dye, FD&C red #40	0	0	0.05	0	0	0
	Dye, FD&C blue #1	0	0	0	0	0.05	0
	Total	1000	4000	1000.0537	4000	1000.0612	4000
	LBI (% compound)			0.00037		0.00112	

### LBI-Administered *Mertk^−/−^Cx3cr1^GFP/+^Ccr2^RFP/+^* Mice Showed Suppression of CCR2-RF-P–Positive Cells in Degenerated Retina

Although there are no CCR2-RFP–positive cells in *Mertk^+/+^Cx3cr1^GFP/+^Ccr2^RFP/+^* mice that do not show RD (Supplementary Fig. S1B), these cells increasingly expand in *Mertk^−/−^Cx3cr1^GFP/+^Ccr2^RFP/+^* mice that develop RD.^21^ The LBI-high mice were provided the supplemented diet from POD 28 to 56 (4 weeks administration). CCR2-RFP–positive cells were counted using retinal sections (Fig. 2). Control diet-fed *Mertk^−/−^Cx3cr1^GFP/+^Ccr2^RFP/+^* mice showed increases in the CCR2-RFP–positive cell count and migration of CX3CR1-GFP–positive cells to the subretinal space (space between the photoreceptor outer segment and RPE) (Fig. 2A 1–3). This retinal phenotype is typical of *Mertk^−/−^Cx3cr1^GFP/+^Ccr2^RFP/+^* mice.^21^ *Mertk^−/−^Cx3cr1^GFP/+^Ccr2^RFP/+^* mice fed the LBI-high feed showed fewer CCR2-RFP-positive cells (Fig. 2B-2, B-3, and C) than the control, indicating suppression of monocyte-derived macrophage invasion from retinal vessels. The number of nuclei in the ONL of the cell body of photoreceptor cells were counted. At POD 56, LBI-high *Mertk^−/−^Cx3cr1^GFP/+^Ccr2^RFP/+^* mice showed marginally ameliorated but almost the same number of ONL nuclei when compared with the control group, indicating LBI administration did not block the initiation of RD (Fig. 2D). Retinal sections of *Mertk^+/+^Cx3cr1^GFP/+^Ccr2^RFP/+^* (without RD) and wild type (WT; B6) mice are shown as negative controls (Supplementary Fig. S1).

### LBI Treatment Suppressed CCR2-RFP But Not CX3CR1-GFP Expression in Retinal Flat Mount

Retinal flat mounting of LBI-high, LBI-low, and control group *Mertk^−/−^Cx3cr1^GFP/+^Ccr2^RFP/+^* mice was performed. Retinal flat mounting of *Mertk^+/+^Cx3cr1^GFP/+^Ccr2^RFP/+^* mice (no RD) is shown as a negative control (Fig. 3A). In *Mertk^+/+^Cx3cr1^GFP/+^Ccr2^RFP/+^* mice, only CX3CR1-GFP–positive microglia were observed (Fig. 4A). Control-feed administered (4-week administration, POD 56) *Mertk^−/−^Cx3cr1^GFP/+^Ccr2^RFP/+^* mice showed marked increase in Ccr2-RFP expression, indicating monocyte-derived macrophage infiltration to the retina (Figs. 3B and D). In contrast, LBI-high and -low administered (4-week administration) *Mertk^−/−^Cx3cr1^GFP/+^Ccr2^RFP/+^* mice showed a decrease in CCR2-RFP expression compared with the control, indicating suppression of monocyte-derived macrophage infiltration to the retina (Figs. 3C, E, and F). Separate CCR2-RFP and CX3CR1-GFP signal images of *Mertk^+/+^Cx3cr1^GFP/+^Ccr2^RFP/+^* mice along with those of control, LBI-high, and LBI-low *Mertk^−/−^Cx3cr1^GFP/+^Ccr2^RFP/+^* mice retinal flat mounts are shown in Supplementary Figure S2.

### LBI Administration Suppressed CX3CR1-GF-P– and CCR2-RFP–Positive Cell Migration to the Subretinal Space

RPE flat mounting was performed to observe the RPE and subretinal space. RPE flat mount of *Mertk^+/+^Cx3cr1^GFP/+^Ccr2^RFP/+^* mice (no RD) showed no CCR2-RFP–positive cells (Fig. 4A). CX3CR1-GFP–positive cells were observed mainly at the OD, and only faint CX3CR1-GFP expression was observed in the RPE and subretinal space (Fig. 4A). Control *Mertk^−/−^Cx3cr1^GFP/+^Ccr2^RFP/+^* mice (POD 56) showed a cluster of CX3CR1-GFP- and CCR2-RFP-positive cells in RPE flat mount, indicating migration of CX3CR1-GFP– and CCR2-RFP–positive inflammatory cells to the subretinal space (Fig. 4B and D). LBI-high *Mertk^−/−^Cx3cr1^GFP/+^Ccr2^RFP/+^* mice (POD 56) showed a decrease in the number of CX3CR1-GFP– and CCR2-RFP–positive cells in RPE flat mount when compared with the control group, indicating that the LBI treatment prevented CX3CR1-GFP– and CCR2-RFP–positive inflammatory cells from migrating to the subretinal space (Fig. 4C and E). Statistical data of CCR2-RFP– and CX3CR1-GFP–positive cell counts are shown in Figures 4F and G. Furthermore, CCR2-RFP– and CX3CR1-GFP–positive cell counts in RPE flat mounts were significantly decreased in the LBI-high and LBI-low groups when compared with the control group. Separate CCR2-RFP or CX3CR1-GFP signal images of RPE flat-mounts for control, LBI-high, and LBI-low *Mertk^−/−^Cx3cr1^GFP/+^Ccr2^RFP/+^* mice are shown in Supplementary Figure S3.

### LBI-Administered *Mertk^−/−^Cx3cr1^GFP/+^Ccr2^RFP/+^* Mice Showed Attenuation of RD at POD 84

LBI-high *Mertk^−/−^Cx3cr1^GFP/+^Ccr2^RFP/+^* mice fed the supplemented feed for 4 weeks (POD 56) showed only minor difference in photoreceptor nuclei count compared with the control group (Fig. 2D). Subsequently, the mice were fed LBI-supplemented or control feed for 8 weeks. Control feed-administered *Mertk^−/−^Cx3cr1^GFP/+^Ccr2^RFP/+^* mice (POD 84) showed severe RD (Figs. 5A and D). Conversely, LBI-high *Mertk^−/−^Cx3cr1^GFP/+^Ccr2^RFP/+^* mice showed preservation of photoreceptor nuclei count compared with the control group (Figs. 5C and F). LBI-low *Mertk^−/−^Cx3cr1^GFP/+^Ccr2^RFP/+^* mice showed slightly severe RD compared with the LBI-high group, but the RD was significantly attenuated when compared with that in the control group (Figs. 5B and E). The entire (from the OD to the ciliary body) inferior retina of the control, LBI-high, and LBI-low *Mertk^−/−^Cx3cr1^GFP/+^Ccr2^RFP/+^* mice are shown in Figures 5D to 5F. LBI-high and -low *Mertk^−/−^Cx3cr1^GFP/+^Ccr2^RFP/+^* mice retained the ONL when compared with the control *Mertk^−/−^Cx3cr1^GFP/+^Ccr2^RFP/+^* mice (Fig. 5G).

### LBI Administration Decreased *Ccl2* and *Ccr2* RNA Expression in *Mertk^−/−^Cx3cr1^GFP/+^Ccr2^RFP/+^* Mice

Retinal RNA levels of *Mertk^+/+^Cx3cr1^GFP/+^Ccr2^RFP/+^* mice were measured as the negative control. At the age of 4 weeks, *Mertk^−/−^Cx3cr1^GFP/+^Ccr2^RFP/+^* mice, which did not show significant RD, showed slightly increased RNA levels of *Ccl2*, *Ccr2*, *Cx3cl1*, and *Cx3cr1* compared with *Mertk^+/+^Cx3cr1^GFP/+^Ccr2^RFP/+^* mice. Control, LBI-high or -low feed was provided to 42 in *Mertk^−/−^Cx3cr1^GFP/+^Ccr2^RFP/+^* mice from POD 28. RNA samples were harvested from the retinas of each group. RNA levels of *Ccl2* and *Ccr2* in the LBI-high and -low groups were decreased compared with those in the control group (Fig. 6, *upper panels*). In contrast, RNA levels of *Cx3cl1* and *Cx3cr1* were not decreased in the LBI-high and -low groups compared with those in the control group (Fig. 6, *lower panels*).

### LBI Administration Preserved Retinal Function in RD

Finally, retinal function was assessed by ERG (Fig. 7). Although there was no significant difference between control or LBI-high–treated *Mertk^−/−^Cx3cr1^GFP/+^Ccr2^RFP/+^* mice in a-wave amplitude (Fig. 7B), there was a significant preservation of b-wave amplitude in LBI-high–treated *Mertk^−/−^Cx3cr1^GFP/+^Ccr2^RFP/+^* compared with that in control mice (Fig. 7C), indicating that LBI treatment contributed to retinal function preservation in RD.

## Discussion

In this study, LBI was administered to *Mertk^−/−^Cx3cr1^GFP/+^Ccr2^RFP/+^* mice. LBI-administered *Mertk^−/−^Cx3cr1^GFP/+^Ccr2^RFP/+^* mice showed a decrease in CCR2-RFP–positive macrophage infiltration to the retina and attenuation of RD. Infiltrated macrophages have been reported to cause harmful inflammation to the central nervous system, including the retina.^25^^,^^26^ To maintain photoreceptors, macrophage infiltration to the retina should be controlled. However, the method or mechanism to prevent harmful inflammation in the retina is currently unknown. We previously reported that minocycline, a semisynthetic, broad-spectrum tetracycline antibiotic, showed therapeutic effect and suppression of CCR2-RFP–positive cell infiltration to the retina in *Mertk^−/−^Cx3cr1^GFP/+^Ccr2^RFP/+^* mice.^7^ Minocycline is frequently used against microbial infections in humans worldwide. During short-term administration, minocycline has limited concerns for adverse effects. However, the life-long administration of minocycline has been continuously raising concerns regarding its side effects involving the central nervous system, such as dizziness, vertigo, ataxia, and tinnitus. Thus, the discovery of effective drug candidates for the prevention of retinal inflammation with fewer side effects has become a focus in ophthalmology and biomedical research. LBI has been administered to patients in Japan for almost 50 years; no remarkable side effects have been reported. Presumably, the strongest point for LBI administration is safety. The adequate LBI dosage for RD treatment is unknown. The permitted LBI dosage is 300 to 600 µg/day, which is 5 to 10 µg/kg for a patient weighing 60 kg. In this study, two types of LBI-containing mice foods were prepared. The mice were fed ad libitum; therefore, the accurate dosage administered could not be measured. If mice eat 2 g food/day, the dosage is approximately 20 µg/kg (LBI-low) or 60 µg /kg (LBI-high), which is 2 to 6 times higher than the human dose. A higher dose seemed to be beneficial for maintaining the photoreceptors in RD. In retinitis pigmentosa, an increased urinary iodine concentration is significantly associated with decreased central foveal swelling in eyes with cystoid macular edema.^27^ This finding could be attributed to the maintenance or recovery of the tight junctions. Iodine promotes tight junction integrity between endothelial cells^28^^,^^29^ and the RPE.^18^ In this study, the number of invaded CCR2-RFP–positive cells number decreased in the LBI-supplemented group. This could be attributed to the maintenance of tight junctions, which act as a blood–retinal barrier blocking the invasion of inflammatory cells into the retina.

However, the toxicity of LBI is unknown. There are several dietary sources of iodine in nature. Representative iodine sources include iodized salt, seafood (such as fish, seaweeds, and shellfish), dairy products, and eggs. As long as the animals received enough iodine and the plants were grown in iodine-rich soil, a sufficient dietary iodine intake is achieved.^30^ Thus, iodine supplementation for patients with RD should be considered. The optimal amount of LBI administration that will reach the intended target tissue, including the retina and choroid, should be evaluated.

In conclusion, oral LBI administration to *Mertk^−/−^Cx3cr1^GFP/+^Ccr2^RFP/+^* mice suppressed CCR2-RFP–positive inflammatory cell infiltration in the retina and subretinal space; RD was also attenuated by LBI administration. Additionally, retinal RNA levels of *Ccl2* and *Ccr2* were suppressed by LBI administration. Corroborating with the previously reported in vitro study,^18^ LBI showed anti-inflammatory properties in vivo in our study. Based on our results, we propose life-long LBI administration as a possible therapeutic option for RD. Further elucidating the underlying mechanism of action and efficacy of LBI would be needed in the future for its large-scale application in the clinical setting.

## Supplementary Material

Supplement 1

Supplement 2

Supplement 3
