# Supplementary figures and images for: Effect of Lecithin-Bound Iodine Treatment on Inherited Retinal Degeneration in Mice
Source: Transl Vis Sci Technol. 2021 Nov 9;10(13):8. doi: 10.1167/tvst.10.13.8 (PMC8590179; doi:10.1167/tvst.10.13.8)

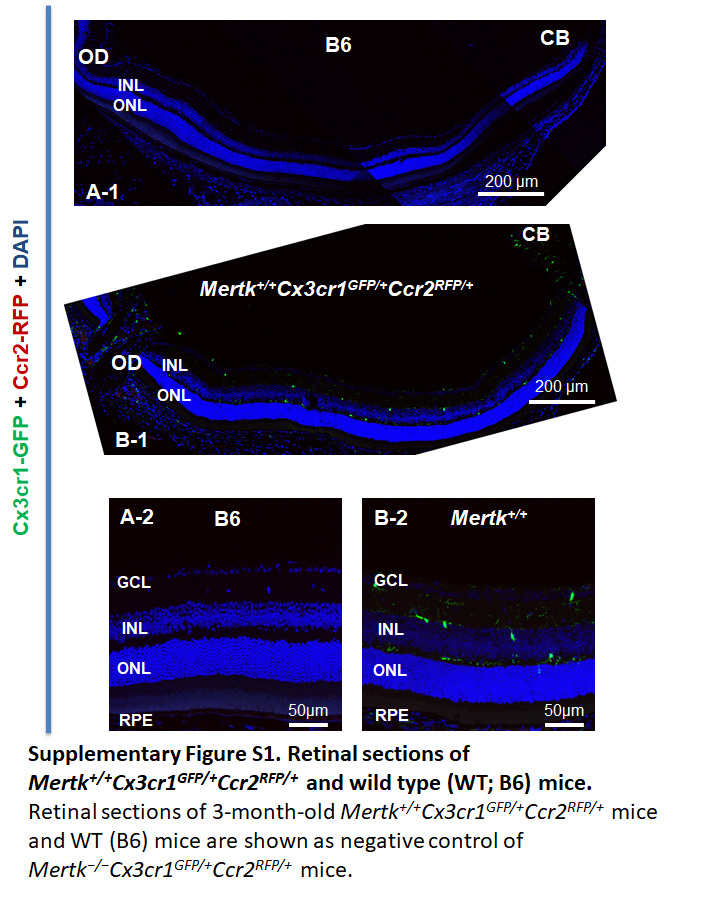

Supplement: Supplement 1 [file tvst-10-13-8_s001.jpg]

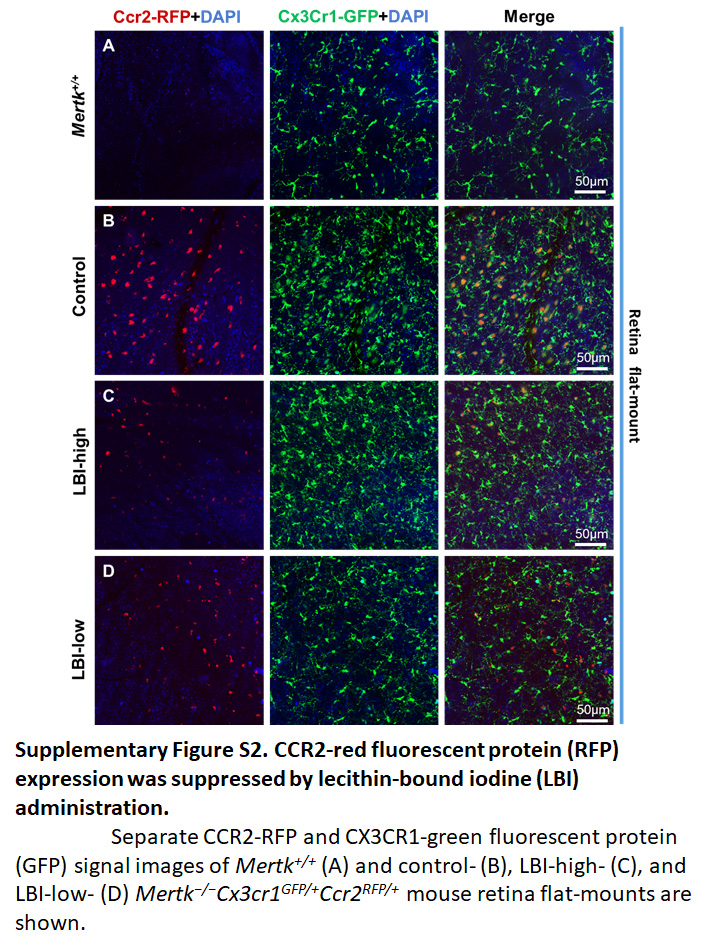

Supplement: Supplement 2 [file tvst-10-13-8_s002.jpg]

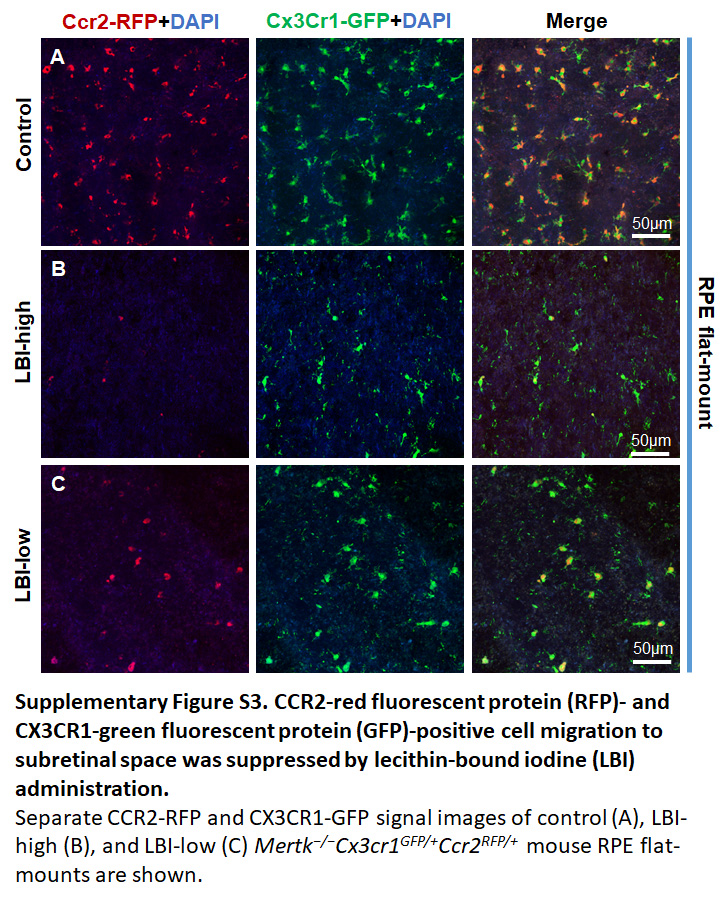

Supplement: Supplement 3 [file tvst-10-13-8_s003.jpg]
